# COE targets EphA2 to inhibit vasculogenic mimicry formation induced by hypoxia in hepatocellular carcinoma

**DOI:** 10.3389/fphar.2024.1421470

**Published:** 2024-07-10

**Authors:** Jue Chen, Shu-Ying Dai, Su Wu, Meng-Ke Wu, Ke-Ke Yu, Jun-Chi Liu, Jia-Yu Chang, Yan-Qing Liu

**Affiliations:** ^1^ Department of Oncology, Affiliated Hospital of Yangzhou University, Yangzhou, China; ^2^ Department of Integrated Traditional Chinese and Western Medicine, Medical College of Yangzhou University, Yangzhou, China; ^3^ The Key Laboratory of Syndrome Differentiation and Treatment of Gastric Cancer of the State Administration of Traditional Chinese Medicine, Yangzhou University, Yangzhou, China; ^4^ Jiangsu Provincial Key Laboratory of Integrated Traditional Chinese and Western Medicine for Prevention and Treatment of Geriatric Diseases, Yangzhou, China

**Keywords:** HCC, vasculogenic mimicry (VM), *Celastrus orbiculatus* extract (COE), EphA2, hypoxia

## Abstract

**Background:**

Vasculogenic Mimicry (VM) can reduce the efficacy of anti-angiogenesis and promote distant metastasis in hepatocellular carcinoma (HCC). Our previous studies have found that *Celastrus orbiculatus* extract (COE) can inhibit the VM formation in HCC by reducing EphA2 expression. However the underlying mechanism related to EphA2 in VM formation is unclear.

**Purpose:**

This study aimed to confirm that EphA2 is one of the potential targets of COE, and to explore the effect of EphA2 in VM formation in hypoxia context in HCC.

**Methods:**

TCM Systems Pharmacology database and proteomics analysis were used to explore the key targets of COE in HCC treatment. CD31-PAS double staining and VE-CAD staining were used to indicate vasculogenic mimicry. The localization of EphA2 and VE-CAD was examined through fluorescent microscopy. CCK8 assay, cell invasion assay, and tube formation assay were used to indicate the formation of VM under hypoxic conditions. The regulatory relationship of EphA2 upstream and downstream molecules were evaluated through COIP and Western Blot. The nude mouse xenograft tumor models were used to observe the VM formation after knocking down or overexpressing EphA2.

**Results:**

EphA2 is identified to the target of COE, and the driving gene of HCC. In HCC surgical specimens, EphA2 expression is closely associated with the VM formation of HCC. COE-regulated EphA2 is involved in hypoxia-induced VM formation in HCC cells in vitro. EphA2 is regulated by HIF directly or indirectly by C-MYC. Overexpression of EphA2 can promote the VM formation of HCC in nude mice, while knocking down EphA2 can inhibit the VM formation.

**Conclusion:**

EphA2, as a target of COE, plays a crucial regulatory role in the formation of vasculogenic mimicry in HCC, involving upstream HIF/MYC transcriptional promotion and downstream PI3K/FAK/VE-CAD expression regulation.

## 1 Introduction

Hepatocellular carcinoma (HCC) is a highly malignant tumor with a rich blood supply. Antiangiogenic drugs such as Bevacizumab and Ramucirumab, which target vascular endothelial growth factor and its receptor, and Sorafenib, Donafenib, and Lenvatinib, which target tyrosine kinase receptor, are the leading choices for first-line treatment of unresectable HCC. However, their objective responding rates are low, and some patients with effective initial treatment soon develop drug resistance and disease progression (Ribatti et al., 2019; Xue et al., 2019; Yao et al., 2023), mainly due to the heterogeneity of HCC blood vessels. HCC cells achieve material exchange between the internal and external environment of the tumor through the endothelial-dependent vessel (EDV), mosaic vessel (MV), and vasculogenic mimicry (VM). VM is simulated blood vessels formed independently by highly invasive tumor cells in the areas away from endothelial-dependent vessels, and there is accumulating evidence indicating that VM is one of the key factors contributing to the failure of anti-angiogenic therapy (Simizu, 2022). Therefore, it is of great significance to conduct in-depth studies on the mechanism of VM formation and intervention strategies to improve the anti-angiogenic efficacy for the treatment of HCC.

Essentially, VM is a phenotypic change in tumor cells to adapt to the barren survival environment, in which hypoxia is the most important environmental factor contributing to the deterioration of the tumor cell phenotype. Prior studies demonstrated that VM is mostly found in the central hypoxic regions of solid tumors. Subsequent findings revealed that hypoxia-induced epithelial mesenchymal transformation of tumor cells and stem-like differentiation of tumor cells promoted VM formation (Yang et al., 2021; Tang et al., 2023). Although anti-angiogenic drugs targeting the endothelium can inhibit the formation of EDV and promote the normalization of its structure and function, this therapeutic strategy does not ameliorate hypoxia within tumor tissues, and instead, it exacerbates the hypoxia in the center of the tumor and promotes VM formation. The formation of VM suggests the resistance of tumor cells to traditional treatments, because VM is the channel connection of the internal and external tumor environments which makes tumor cells more susceptible to form distant metastasis. Therefore, investigating the regulatory mechanism of VM formation in the specific microenvironment of hypoxia would help develop novel therapeutic strategies and drugs.

Some natural medicines and their extracts, such as Huaier granules and Icaritin capsules, are known to have anti-HCC effects (Li et al., 2022; Tang et al., 2023). In our previous study, we found that *celastrus orbiculatus* extract (COE) inhibited the proliferation and invasion of HCC cells and blocked VM formation both *in vitro* and *in vivo* of HCC cells (Chu et al., 2021a). Through proteomics technology, we obtained the differential proteins after COE action on MHCC97-H cells, among which the erythropoietin-producing hepatocyte receptor A2 (EphA2) has received our attention because of its extensive involvement in the malignant biological behaviors of HCC. Blockades of EphA2 (EphA2-specific antagonists) affect VM formation in HCC cells. It has been reported that EphA2 is involved in VM formation in various tumors, but its role and the related mechanisms in VM formation in HCC are elusive. Whether EphA2 is involved in VM formation in a hypoxic environment and its upstream and downstream regulatory mechanisms are not clear.

In this study, we aimed to find out how EphA2, one of the COE targets, regulates VM formation under the hypoxic microenvironment in HCC. The specific molecular mechanism of EphA2 in regulating VM formation in the hypoxic microenvironment of HCC cells was revealed by various experiments. The results of this study will provide a new strategy for improving the therapeutic response of HCC.

## 2 Materials and methods

### 2.1 Screening of components and targets of *Celastrus orbiculatus*

We used Pub Chem (https://pubchem.ncbi.ni.nih.gov) to screen out the canonical SMILES number of the chemical constituents in the stems of *Celastrus orbiculatus*. With the help of the Swiss ADME platform, the GI absorption score was “high,” and the drug-likeness was screened by at least two “yes” for secondary screening. The canonical SMILES of the screened compounds was input into the Swiss TargetPrediction database (http://www.swisstargetprediction.ch/) to predict the targets of each chemical component. After data statistics and removal of duplication, the target protein information related to the active components of *Celastrus orbiculatus* was obtained. Cytoscape software was used to draw the network diagram of drug component-target interaction of *Celastrus orbiculatus*.

### 2.2 Collection of HCC-related target genes

In the GeneCards (http://www.genecards.org/) database and OMIM (https://www.omim.org) database, “hepatocellular carcinoma” was used as the keyword, and the attribute was set to “*Homo sapien*” for retrieval. The information of HCC-related targets was obtained, and the potential targets of HCC were obtained after data deduplication.

### 2.3 Determination of intersection target

The screened targets of the active components of *Celastrus orbiculatus* and HCC-related targets were imported into the Weishengxin online cloud platform (https://www.bioinformatics.com.cn/) for comparative analysis to draw the Venn diagram, and the intersection targets of the active components of *Celastrus orbiculatus* and HCC were obtained.

### 2.4 Patient and HCC samples

HCC tissues were obtained from the surgery in patients with HCC at the Second People’s Hospital of Taizhou (Jiangsu, China). This study was approved by the Ethics Committee of the Second People’s Hospital of Taizhou No. TZEYLL20180301.

### 2.5 Cell lines and culture conditions

Two human HCC cell lines, MHCC97-H and HepG2, were purchased from Zhong Qiao Xin Zhou Biotechnology Company (Shanghai, China). The cells used in this study were cultured in Dulbecco’s modified Eagle’s medium (DMEM) (Gibco, Grand Island, NY, United States). The above cells were cultured in an incubator containing 10% fetal bovine serum (FBS) (Gibco, Grand Island, NY, United States), 100 u/mL penicillin, 100 mg/mL streptomycin, and 2 mmol/l L-glutamine as well as 37°C, 95% humidity and 5% CO_2_ conditions. Subsequently, the cells were grouped and subjected to knockdown groups (Ctrl, Hypoxia, Hypoxia + COE 80 μg/mL, Hypoxia + EphA2 siRNA) and overexpression groups (Hypoxia, Hypoxia + EphA2 OE, Hypoxia + COE μg/mL, Hypoxia + COE μg/mL + EphA2 OE) of EphA2 experiments.

### 2.6 Source of COE

*Celastrus orbiculatus* Thunb (Sapindus order Celastraceae, www.theplantlist.org) was planted in Guangxi Province (south-west areas of China), harvested and processed by Zhixin Pharmaceutical Company (SN: 170812, Guangzhou, China). Herb identification and extraction procedure has been described previously (Qian et al., 2012; Jiang et al., 2019). Briefly, COE was dissolved in dimethyl sulfoxide (DMSO) as a 0.016 g/mL stock solution. When used, the stock solution was diluted into different working concentrations using culture medium. In this study the final concentration of DMSO in working fluid was less than 0.01% (Chu et al., 2021a).

### 2.7 Hypoxia treatment

Hypoxia condition was mimicked for *in vitro* cell culture by flushing out air and replacing with 1% O_2_/5% CO_2_ for 2 min at 40 L/min rate inside a modulator incubator chamber of 20 L capacity. Experiments with paired normoxia (20% O_2_/5% CO_2_) and hypoxia samples were incubated in the same 37°C incubator.

### 2.8 VM formation observation

The experimental procedure was described previously (Chu et al., 2021b). Briefly, 24-well tissue culture plates were taken and each well was coated with 300 µL of growth factor-reduced matrix gel (Corning Co., Shanghai, China) and cured at 37°C for 30 min. Cell suspension (2 × 10^5^ cells/well) was added to the surface of the matrix gel and incubated at 37°C for 12 h. Cells were photographed using an Olympus CKX41 inverted microscope (Olympus, Tokyo, Japan).

### 2.9 HE staining

Paraffin-embedded HCC tissue specimens were routinely sectioned, baked, deparaffinized, and rehydrated. Hematoxylin staining of the nucleus for 5 min, and eosin staining of the cytoplasm for 5 min. After washing, dehydrating with ethanol, and clearing with xylene for 2 min, the sections were sealed with neutral resin. The prepared sections were placed under the microscope for observation and image acquisition.

### 2.10 Immunohistochemistry (IHC) and PAS staining

HCC paraffin-embedded tissue was sectioned at 4-μm thick, dewaxed and hydrated. Citrate buffer (pH 7.8, 0.1 M) was used for antigen repair for 20–30 min. Endogenous peroxidase activity was blocked by uniform coverage with endogenous peroxidase blocking solution (Solarbio, Beijing, China) for 10 min, and the sections were blocked with normal goat serum for 30 min. Sections were incubated with primary antibody overnight at 4°C, washed with PBS, and incubated with secondary biotinylated goat anti-rabbit IgG for 60 min. After color development by 3,3′-diaminobenzidine, the process entered into subsequent PAS staining. PAS staining was performed using a PAS staining kit (SN: DG0005, Leagene Biotechnology Co., Ltd., Beijing, China). Briefly, after the DAB reaction, the slices were treated with 0.5% periodate solution for 10 min, rinsed in distilled water for 3 min, and stained in Schiff’s solution for 20 min before hematoxylin re-staining, dehydration, clearing and sealing.

### 2.11 Cell proliferation test

Cell proliferative activity was assessed using a cell counting kit-8 (CCK-8) (BBI Life Sciences, Shanghai, China). The complete medium was made into single cell suspension, adjusted to a concentration of 1×10^5^ cells/mL, and inoculated into 96-well culture plates at 100 µL per well. After 1, 2, 3, and 4 days of incubation, 10 μL of CCK-8 assay solution was added to each well at the indicated times and incubated in an incubator with 37°C for 1 h. Automated enzyme labelling instrument (BioTek Instruments Inc., Vermont, United States) is used to measure the optical density (OD) read at 450 nm wavelength.

### 2.12 Cell invasion assay

Cell invasion assays were processed as previously described (Li et al., 2019). Logarithmic growth phase HCC cells were taken in serum-free medium to make single cell suspension of 5 × 10^5^ cells/mL, and 100 µL/well was inoculated in the upper chamber of the Transwell. In the lower chamber, 600 µL of DMEM medium containing 20% FBS was added to each well. After 24 h of incubation in the cell culture chamber, non-invasive cells were removed, and the migrated cells on the bottom surface of the membrane were fixed with formaldehyde, stained with 0.1% crystal violet solution for 30 min, and counted under an Olympus CKX41 inverted microscope (Olympus, Tokyo, Japan) in three different fields of view randomly selected from each well (×400 magnification).

### 2.13 Western Blot analysis

The experimental procedure was as described previously (Chen et al., 2020). Briefly, total cellular proteins were extracted with RIPA lysate, protease inhibitor and phosphatase inhibitor, and protein concentration was determined by BCA method. It was separated by polyacrylamide gel electrophoresis and transferred to PVDF membrane. 5% skimmed milk was used for blocking at room temperature for 1 h. Primary antibody was added and incubated overnight at 4°C. After washing the membrane, secondary antibody was added and incubated at 37°C for 1 h. Protein bands were detected by gel imaging analysis system. The bands were analyzed in grey scale according to the imaging system software ImageJ 1.54f (National Institutes of Health), and the protein expression levels of the target bands were analyzed using GAPDH as an internal reference. Each experiment was repeated three times. The sources and dilutions of the antibodies are shown in Supplementary Table S1.

### 2.14 Fluorescent staining

HCC paraffin-embedded tissue sections were used. Rabbit anti-human fluorescent primary antibody was used to stain the sections in darkness for 30 min. The second drop of co-stained VE-Cad antibody diluted at 1:1,000 was added, and then FITC-labelled goat anti-rabbit IgG fluorescent antibody was added to stain the sections at 37°C for 30 min. The nuclei of the cells were stained by DAPI, and the sections were sealed by fluorescent sealing solution, stored at 4°C overnight. Then localization was observed by laser confocal microscopy. Co-localization analysis was performed by qualitative and quantitative analyses. Qualitative analysis was performed by observing the overlap (co-occurrence) of two different fluorescent molecules clusters in pixel space, and quantitative analysis was performed by describing the variable relationship (correlation) in a statistical manner (Pearson’s correlation coefficient) via ImageJ 1.54f (National Institutes of Health) software.

Interpretation of results of Pearson’s correlation coefficient between fluorescence signals of cell surface molecules are as follow: the correlation coefficient R between the two groups of observed parameters in this study was assessed via Pearson’s correlation analysis (ImageJ 1.54f and GraphPad 8.0.2). Briefly, ImageJ 1.54f (National Institutes of Health) software was used to analyze and calculate the R value. R values were interpreted according to the following criteria: R values range −1∼ +1, *R* = −1, completely negative linear relationship; −1 < *R* ≤ −0.7, strong negative linear relationship; −0.7 < *R* ≤ −0.4, negative correlation; −0.4 < *R* ≤ −0.2, weak negative linear relationship; −0.2 < *R* ≤ +0.2, no significant relationship; +0.2 < *R* ≤ +0.4, weak positive correlation; +0.4 < *R* ≤ +0.7, positive linear relationship; +0.7 < *R* < +1, strong positive linear relationship; *R* = +1, perfect positive linear relationship. We consider r > +0.2 as a positive correlation.

### 2.15 Animals and tumor xenograft assay

The protocol for animal study was the same as previously described (Wu et al., 2020). Briefly, 15 male BALB/c/nu mice, aged 5–8 weeks, weighing 18–20 g, were obtained from the center for Comparative Medicine of Yangzhou University (Yangzhou, Jiangsu, China). The experimental animal license number is SYXK (Su) 2022-0044. The mice were housed in a laminar-flow chamber (SPF grade) with a temperature of 22°C ± 2°C, relative humidity of 55% ± 5%, light-dark cycle of 12 h, and sterility and specific pathogen-free. The protocol complied with internationally recognized guidelines on the use of laboratory animals and has been approved by the Ethics Committee of Yangzhou University (IACUC).

MHCC97-H cells transfected with EphA2 OE/KD were resuspended in 100 mL PBS at a concentration of 1 × 10^7^ cells/mL and injected subcutaneously into the left axilla of nude mice. The tumor-bearing nude mice were marked and the groups were recorded (five mice in each group) in order to observe their growth changes. During the experiment, the long diameter (L), short diameter (W) and height (H) of the graft tumor were measured with a vernier caliper. The tumor volume (V) was calculated as follows: V = 1/2×L×W^2^. The dynamic tumor growth curve was plotted. The nude mice in each group were sacrificed by cervical dislocation on the 28th day after modelling, and the tumor blocks were dissected out and weighed. The tumor blocks were divided and frozen at −80°C, 3% glutaraldehyde fixation and formalin fixation, for pathological and histological staining. All procedures for animal use were in accordance with the Guide for the “Laboratory Animals Care and Use Guidelines” (NIH Publication No. 80-23, Revised 1996) and were performed in accordance with institutional ethical guidelines for animal experimentation.

### 2.16 Chromatin immunoprecipitation (ChIP) technology

Experiments were performed according to the instructions of the EZ-Magna ChIP TMA-Chromatin Immunoprecipitation Kit (Merck Miller Pack Company). The protein EphA2 promoter complex was immunoprecipitated with C-MYC antibody (ab11917, Abcam) with INPUT as positive control and IgG as the negative control. DNA collect by these antibodies was subjected to PCR analysis, followed by sequencing. ChIP primers were designed as in Supplementary Table S2. Amplification of soluble chromatin prior to immunoprecipitation was used as the input control.

### 2.17 Statistical analysis

Continuous variable data was expressed as mean ± standard deviation (mean ± SD). Statistical analysis was performed using GraphPad 8.0.2 (GraphPad Software) statistical software. The continuous variables between the two groups were analyzed by *t*-test, and the dichotomous variables between the two groups were analyzed by Chi-Square test. *p* < 0.05 indicates a statistical difference.

## 3 Results

### 3.1 EphA2 is one of the targets of COE in the treatment of HCC

**FIGURE 1:**
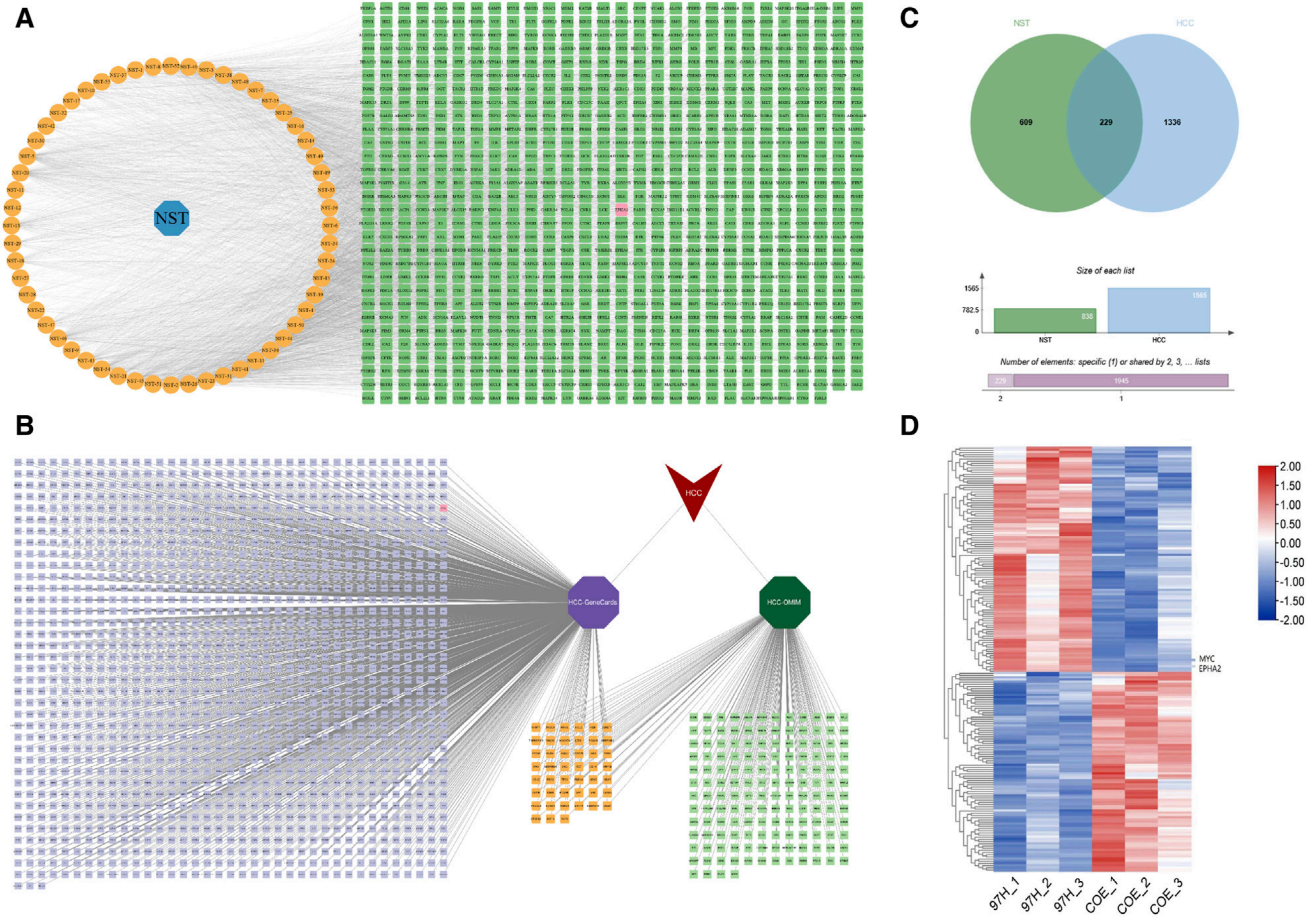
Analysis of “*Celastrus orbiculatus*-active components-HCC related targets” network and common targets. **(A)** Clustering analysis of differentially expressed genes of tissue proteins in patients with HCC. **(B)** Network diagram of *Celastrus orbiculatus*drug-components-target interactions. NST: *Celastrus orbiculatus*; orange nodes: active components of *Celastrus orbiculatus*; green nodes: predicted targets. **(C)** Potential targets of hepatocellular carcinoma in GeneCards and OMIM databases. **(D)** Wayne diagram of *Celastrus orbiculatus*components targets and HCC disease targets.

COE is a mixture of polyterpenoids obtained by ethyl acetate extraction from the stems of *Celastrus orbiculatus* (Feng et al., 2023). We have previously identified the name and structure of the terpenoids contained in COE. In this study, we again screened the chemical constituents in the stems of *Celastrus orbiculatus*. We conducted online analysis through network pharmacology technology. In the end, 56 components of *Celastrus orbiculatus* were obtained, and a cumulative total of 828 drug-gene targets, including EphA2, were obtained (Figure 1A). HCC-related gene targets were downloaded through the GeneCards database and OMIM database, and 1,565 disease gene targets were finally obtained by screening integration and de-emphasis (Figure 1B). The intersection of the two was taken, and a total of 229 potential targets were obtained, including EphA2 (Figure 1C). Finally, on the basis of database analysis, we treated human HCC cells MHCC97-H with COE, performed protein profiling (http://www.proteomexchange.org/, PXD022203), and lastly identified EphA2 as the target of COE action in HCC treatment (Figure 1D).

### 3.2 The increased expression of EphA2 is closely associated with VM formation in HCC

**FIGURE 2:**
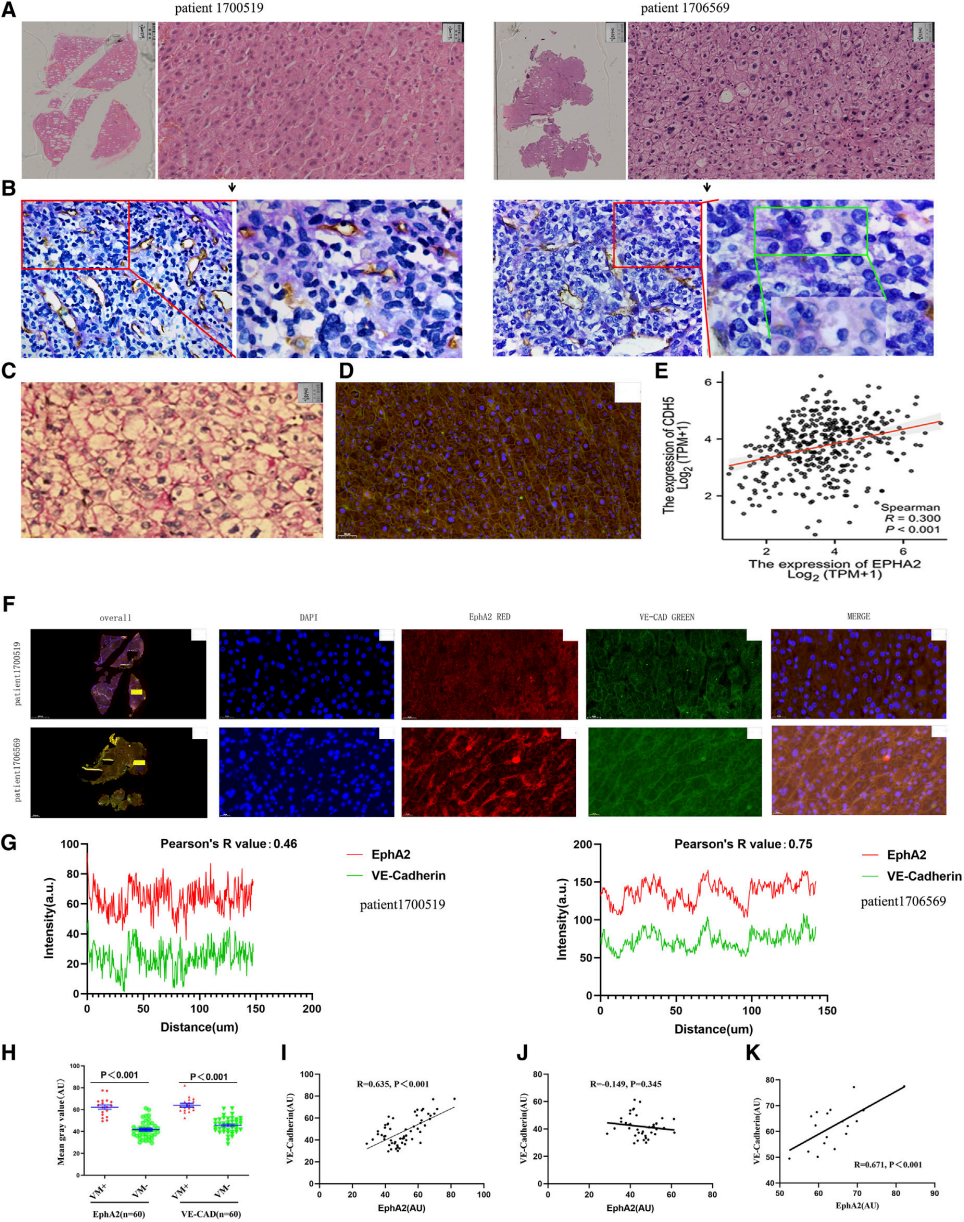
**(A,B)** VM was detected in poorly differentiated HCC away from the EDV region. A HE staining of surgical specimens from two cases of HCC, scale: 50 µm. **(B)** PAS-CD31 staining of consecutive sections with specimens from A. **(C)** PAS staining in poorly differentiated HCC showed a large number of striped purplish-red PAS-positive structures. **(D)** IF staining of VE-CAD in poorly differentiated HCC specimens showed a cord-like staining of the cell membrane, consistent with the location of PAS-positive structures, scale: 50 µm. **(E)** TCGA database confirmed that there was a positive correlation between the expression levels of EphA2 and VE-CAD (CDH5) in HCCs (R = 0.300, *p* < 0.001). **(F,G)** There was a positive correlation between the expression levels of EphA2 and VE-CAD in specimens with VM detected by immunofluorescence (blue: DAPI, red: EphA2, green: VE-CAD, scale: 20 µm, R = 0.75). **(H)** In 60 surgical specimens of liver cancer, the expression levels of EphA2 and VE-CAD with VM were higher than those without VM (n = 60, *p* < 0.001). **(I)** There was a positive correlation between the expression levels of EphA2 and VE-CAD in specimens (r = 0.635, *p* < 0.001). **(J, K)** There was no positive correlation between the expression levels of EphA2 and VE-CAD in HCCs without VM (r = −0.145, *p* > 0.05), There was a positive correlation between the expression levels of EphA2 and VE-CAD in HCCs with VM (r = 0.671, *p* < 0.001).

Among the 60 surgical specimens of resectable HCC, we found VM structures in a total of 18 cases. Comparison by HE staining revealed that VM generally appeared in the region of distant EDV, that is, close to the central region of the tumor. Additionally, HCC cells with VM structures tended to be poorly differentiated (Figures 2A, B). Previous studies have confirmed that VE-CAD is a characteristic phenotypic protein that forms VM in tumor cells (Delgado-Bellido et al., 2023). We found that the staining site and staining intensity were consistent in HCC tissues with VM by PAS staining and VE-CAD immunofluorescence staining (Figures 2C, D). The results suggest that VE-CAD can be used as a characteristic marker of VM in HCC. Through the analysis of the TCGA-LIHC dataset, it was found that the transcription level of EphA2 was positively correlated with VE-CAD (*R* = 0.30, *p* < 0.001, Figure 2E). Double immunofluorescence staining of EphA2 and VE-CAD in HCC specimens with and without VM revealed that there was a significant concordance between EphA2 and VE-CAD in terms of the expression site and expression intensity in HCC tissues with VM structures (Pearson’s R = 0.75). However, there was only slight concordance between the two in HCC tissues without VM structures (Pearson’s R = 0.46, Figures 2F, G). The mean fluorescence intensity (AU) of EphA2 and VE-CAD in the 60 HCC tissues with VM structures was significantly higher than that in HCC without VM (63.39 ± 8.02 VS. 64.39 ± 7.25, *p* < 0.001; 41.75 ± 7.74 VS. 45.75 ± 7.07, *p* < 0.001, Figure 2H). In the 60 HCC tissue specimens, the fluorescence intensity of EphA2 had a positive correlation with VE-CAD (*R* = 0.635, *p* < 0.001, Figure 2I), demonstrating consistency with the results of TCGA database analysis. In particular, the correlation between the two was more pronounced in VM-positive specimens (*R* = 0.671, *p* < 0.001, Figure 2J); whereas this correlation was not demonstrated in HCC tissues without VM structures (*R* = 0.149, *p* = 0.345, Figure 2K). The localization and quantitative relationship between EphA2 and VE-CAD, a characteristic VM protein, revealed that EphA2 was closely associated with the formation of VM in HCC.

### 3.3 COE-regulated EphA2 is involved in hypoxia-induced VM formation in HCC *in vitro*

**FIGURE 3:**
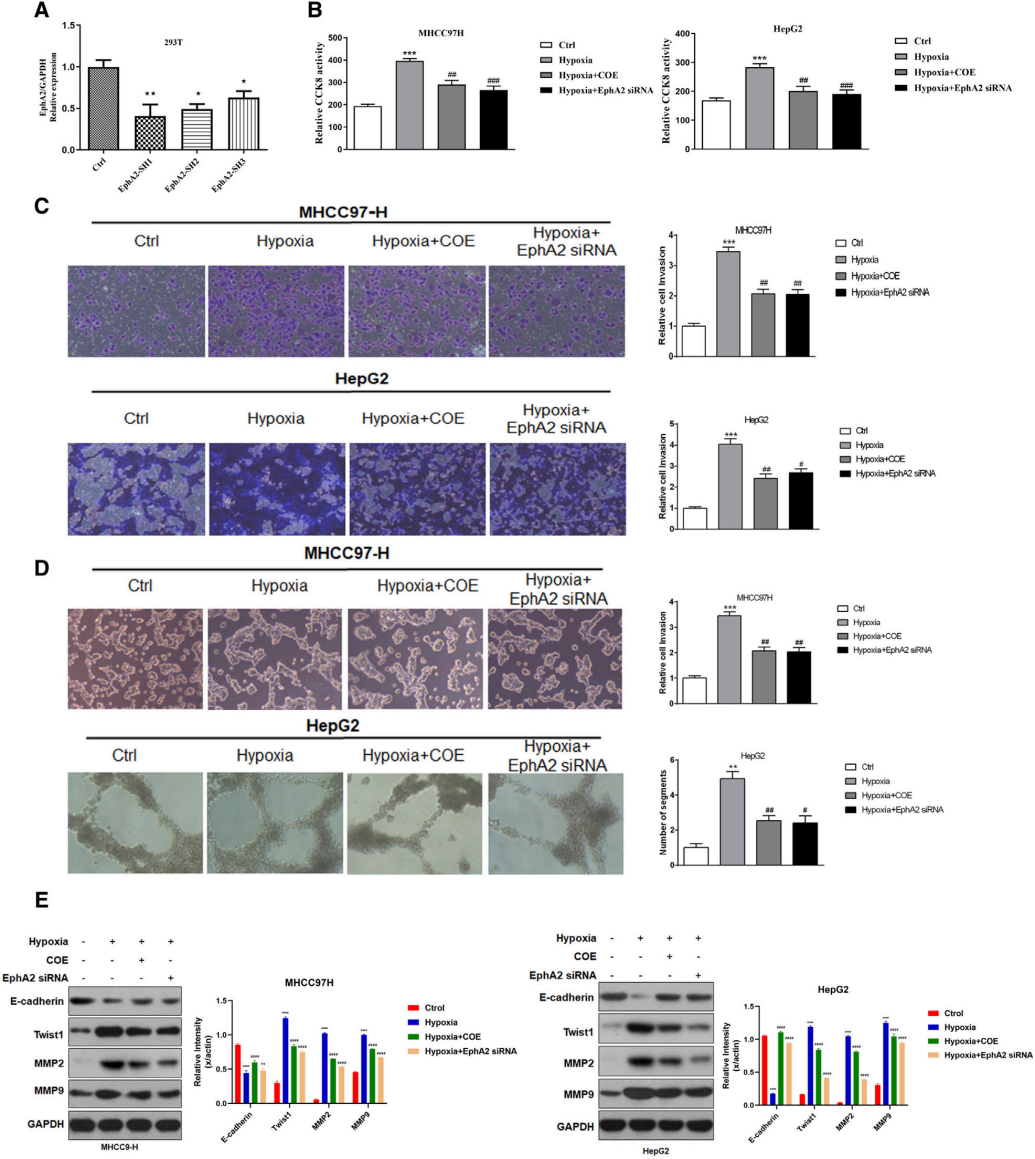
The effect of inhibiting EphA2 expression on hypoxia-induced VM formation in HCC cells. **(A)** EphA2 siRNA was constructed and achieved a significant decrease in its expression. **(B)** Hypoxia increased proliferative activity in HCC cells. ***, hypoxia VS. normoxia (Ctrl), *p* < 0.001. Proliferative activity of HCC cell was suppressed after applying siRNA or COE to inhibit EphA2 expression. ##, siRNA EphA2 VS. hypoxia, COE VS. hypoxia, *p* < 0.001. **(C)** Hypoxia increased the invasive ability of HCC cells, ***, hypoxia VS. Ctrl, *p* < 0.001. HCC cell invasive ability was inhibited after applying siRNA or COE to inhibit EphA2 expression, ##, siRNA EphA2 VS. hypoxia, COE VS. hypoxia, *p* < 0.001. **(D)** Hypoxia promoted VM formation in HCC cells, ***, hypoxia VS. normoxia (Ctrl), *p* < 0.001. Inhibition of EphA2 expression by siRNA or COE inhibited VM formation in HCC cells, ##, siRNA EphA2 VS. hypoxia, COE VS. hypoxia, *p* < 0.001. **(E)** Hypoxia resulted in increased expression of VM-related proteins (MMP2, MMP9 and TWIST) and decreased expression of E-CAD, ***, hypoxia VS. normoxia (Ctrl), *p* < 0.001. VM-related protein expression was altered after inhibition of EphA2 by application of siRNA or COE, ##, siRNA EphA2 VS. hypoxia, COE VS. hypoxia, *p* < 0.001. Hypoxia, COE VS. hypoxia, *p* < 0.001.

Our previous study has confirmed that antagonism of EphA2 signaling can inhibit the formation of VM in HCC cells (Chu et al., 2021b). Another previous study had found that COE inhibited the proliferation of MHCC97-H cells in a time-and concentration-dependent manner, and the half inhibitory concentration after 24 h treatment was 145.88 μg/mL. In order to avoid the cytotoxicity of COE, we chose to use a maximum concentration of 80 μg/mL with a concentration of less than half of the inhibitory concentration in subsequent experiments. In this study, we used siRNA to specifically inhibit the expression of EphA2 to observe the VM formation of HCC cells (Figure 3A). The proliferation and invasion ability of MHCC97-H and HepG2 cells were significantly enhanced under hypoxic conditions (Figures 3B, C), and the tubule formation ability of the HCC cells was significantly increased under three-dimensional conditions (Figure 3D). In contrast, the above hypoxia-induced tumor cell growth were significantly inhibited after siRNA knockdown of EphA2 expression (Figures 3A–C). Previous studies have reported that MMP2, MMP9 and TWIST are important functional proteins involved in VM formation in tumor cells (Yao et al., 2016; Li et al., 2017). In this study, we found that the expression of these functional proteins was elevated after hypoxia, while the expression was suppressed after knockdown of EphA2 (Figure 3E). By adding COE (80 μg/mL), the changes in cell proliferation, invasion, VM formation and expression of VM-related proteins under hypoxic conditions were similar to the effects of EphA2 knockdown. These results suggest that siRNA intervention or inhibition of EphA2 expression by COE could attenuate hypoxia-induced VM formation in HCC.

**FIGURE 4:**
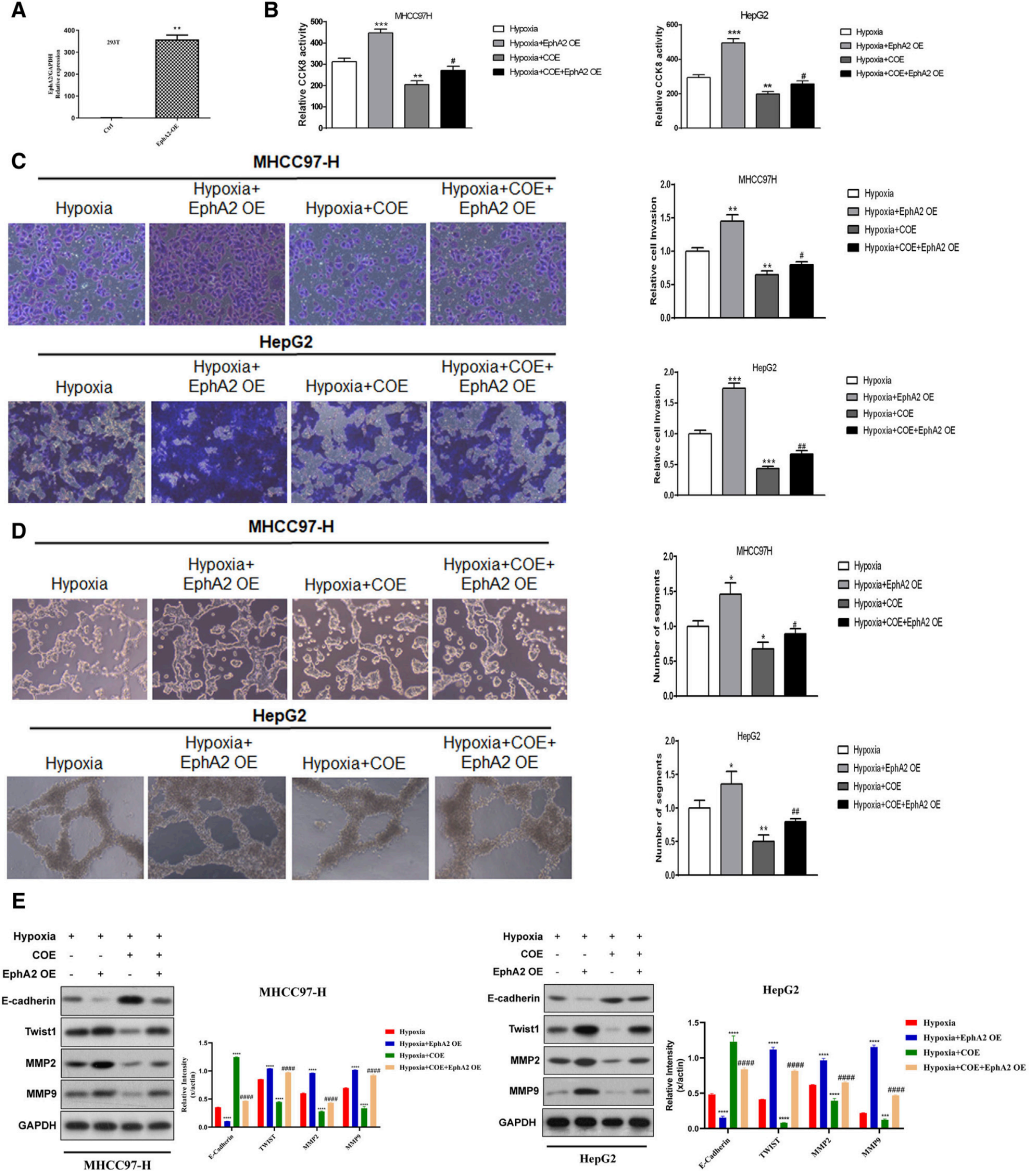
The effect of overexpression of EphA2 on hypoxia-induced VM formation in HCC cells. **(A)** EphA2 overexpression plasmid was constructed and verified in 293T cells. **(B)** Overexpression of EphA2 enhanced hypoxia-induced HCC cell proliferation. COE inhibited HCC cell proliferation promoted by overexpression of EphA2, while overexpression of EphA2 partially reversed COE-induced HCC cell proliferation. ***, hypoxia + COE VS. hypoxia (Ctrl), *p* < 0.001. #, hypoxia + COE + EphA2 OE VS. hypoxia + EphA2 OE, *p* < 0.05. **(C)** Overexpression of EphA2 promoted HCC cell invasion under hypoxia. COE inhibited the enhanced invasion due to overexpression of EphA2, which was partially reversed by overexpression of EphA2. ***, hypoxia + COE VS. hypoxia (Ctrl), *p* < 0.001. ##, hypoxia + COE + EphA2 OE VS. hypoxia + EphA2 OE, *p* < 0.01. **(D)** Overexpression of EphA2 promoted hypoxia-induced VM formation and COE inhibited tubule formation induced by overexpression of EphA2, which was partially reversed by overexpression of EphA2. ***, hypoxia + COE VS. hypoxia (Ctrl), *p* < 0.001. ##, hypoxia + COE + EphA2 OE VS. hypoxia + EphA2 OE, *p* < 0.01. **(E)** Overexpression of EphA2 increased expression of MMP2, MMP9 and TWIST, and decreased the expression of E-CAD. ***, hypoxia VS. normoxia (Ctrl), *p* < 0.001. COE inhibited VM-related protein expression induced by EphA2 overexpression. ##, siRNA EphA2 VS. hypoxia, COE VS. hypoxia, *p* < 0.001.

To further confirm that the regulation of EphA2 by COE was involved in hypoxia-induced VM formation in HCC cells *in vitro*, we overexpressed EphA2 in HCC cells (Figure 4A). The results showed that overexpression of EphA2 further enhanced the proliferation, invasion, VM formation and related protein expression of HCC cells under hypoxic conditions. COE (80 μg/mL) treatment inhibited hypoxia-induced HCC cell invasion and VM formation, but overexpression of EphA2 partially reversed this inhibitory effect of COE (Figures 4B–E). These results suggested that COE regulated the expression of EphA2 and involved in hypoxia-induced VM formation in HCC cells.

### 3.4 EphA2 is regulated by HIF-1a directly or indirectly via C-MYC

**FIGURE 5:**
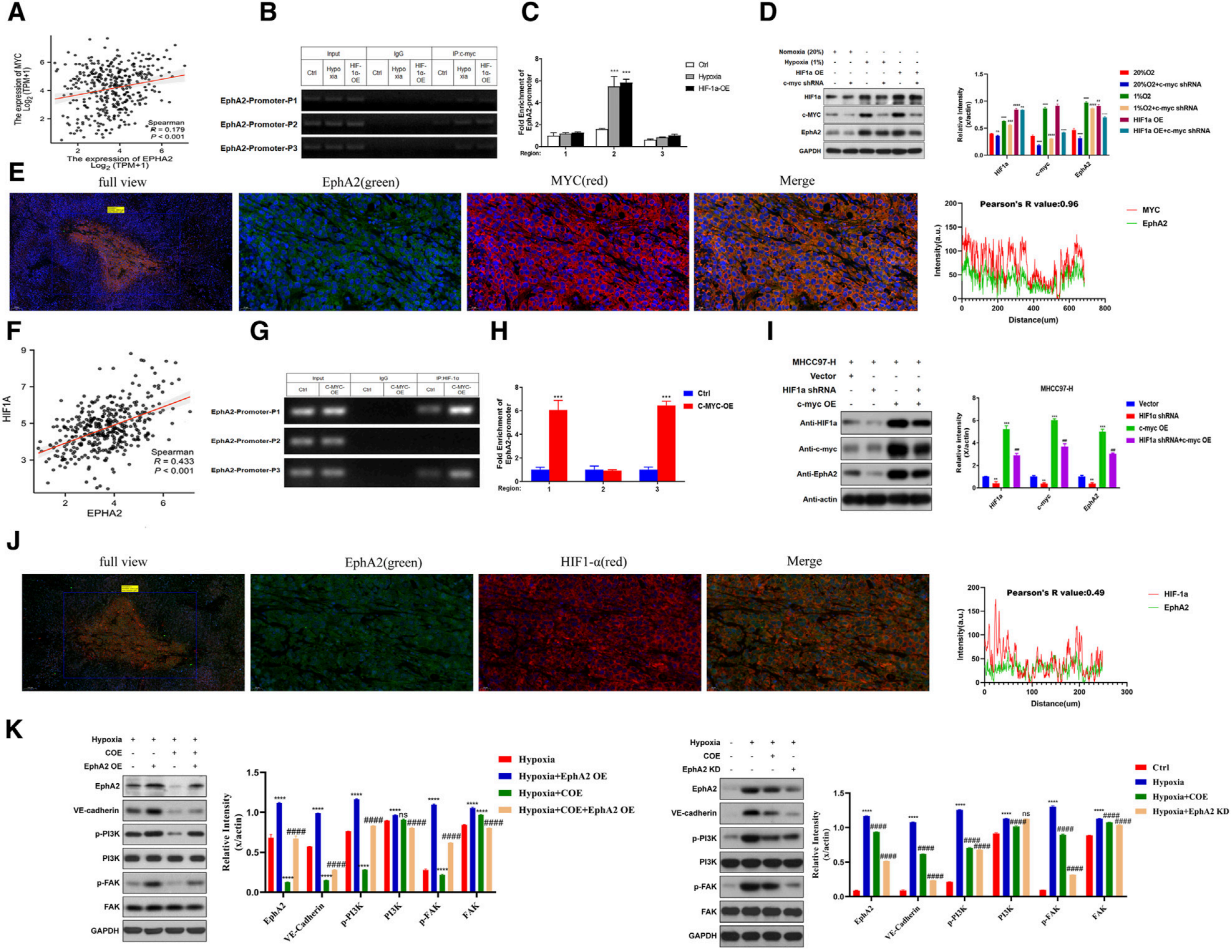
Hypoxia and C-MYC-mediated EphA2 is prominent in the formation of HCC vasculogenic mimicry. **(A)** The TCGA database confirmed positive correlation between EphA2 and MYC expression in HCC (*R* = 0.179, *p* < 0.001). **(B, C)** C-MYC interacts with EphA2 via co-immunoprecipitation (Co-IP) assay. **(D)** Western Blot detecting the protein expression levels of HIF-1a, C-MYC, and EphA2 after Normoxia (20%), Hypoxia (1%), HIF-1a OE, and C-MYC shRNA treatment. **(E)** The relationship between the localization and level of EphA2 and MYC in nude mice HCC specimens detected by immunofluorescence (green: EphA2, red: MYC, *R* = 0.96, scale: 20 um). **(F)** The TCGA database confirmed positive correlation between EphA2 and HIF-1a expression levels in HCC (*R* = 0.433, *p* < 0.001). **(G, H)** HIF-1a interacts with EphA2 via co-immunoprecipitation (Co-IP) assay. **(I)** Western Blot was used to detect the protein expression levels of HIF-1a, C-MYC, and EphA2after Vector, HIF-1a shRNA, and C-MYC OE treatment. **(J)** The relationship between the location and expression of EphA2 and HIF-1a in nude mice HCC specimens was detected by immunofluorescence co-localization (green: EphA2, red: HIF-1a, *R* = 0.96, scale: 20 um). **(K)** Western Blot was used to detect the protein expression levels of EphA2, VE-CAD, p-PI3K, PI3K, p-FAK, and FAK after treatment with hypoxia, COE, and EphA2 (OE/KD).

We found that C-MYC and EphA2 showed consistent downregulation in the differentially expressed proteins in COE treated MHCC97-H cells (Figure 1C). As an important hypoxia-responsive protein, C-MYC participates in the regulation of downstream signaling after receiving HIF-1a transcriptional promotion. Through TCGA database analysis, it was found that C-MYC was linearly correlated with EphA2 (*R* = 0.179, *p* < 0.001, Figure 5A). JASPAR database predicted that the EphA2 promoter region had a corresponding sequence available for C-MYC binding (Sup.). Immunoprecipitation experiments revealed that there was a significant enrichment in the P2 segment of the EphA2 promoter region upon input C-MYC (Figures 5B, C). The expression of C-MYC and EphA2 was upregulated under hypoxia condition and overexpression of HIF-1a in MHCC97-H cells, and the expression of EphA2 was decreased after knockdown of C-MYC under the same conditions (Figure 5D). Additionally, C-MYC co-localized with EphA2 in MHCC97-H nude mouse transplant tumors (*R* = 0.96, Figure 5E). These results suggest that EphA2 is regulated by HIF-1a through C-MYC under hypoxic conditions.

In addition, we found the same positive correlation between EphA2 and HIF-1a by TCGA database (*R* = 0.433, *p* < 0.001, Figure 5F). In co-immunoprecipitation, HIF-1a was found to be able to bind to the EphA2 promoter region (Figures 5G, H), suggesting that HIF-1a could promote the transcriptional expression of EphA2. In MHCC97-H cell line, the protein expression levels of C-MYC and EphA2 decreased after knockdown of HIF-1a, while overexpression of C-MYC significantly upregulated EphA2 protein expression levels (Figure 5I). In the transplanted MHCC97-H tumor tissues of nude mice, immunofluorescence experiments showed mild concordance between EphA2 and HIF-1a in terms of expression location and expression intensity (Pearson’s R value = 0.49, Figure 5J). The above results suggest that EphA2 is regulated by HIF-1a directly or indirectly via C-MYC.

Previous studies have reported that the activation of PI3K/FAK pathway can promote the expression of VE-CAD and participate in the formation of VM in tumor cells (Zhou et al., 2021; Murai and Matsuda, 2023). In this study, we observed that the phosphorylation levels of PI3K and FAK proteins were upregulated after overexpression of EphA2 under hypoxic conditions, and the expression of VE-CAD was increased. COE can inhibit the phosphorylation of PI3K and FAK proteins caused by EphA2 overexpression and inhibit the expression of VE-CAD (Figure 5K). Correspondingly, the phosphorylation of PI3K and FAK was inhibited and the expression of VE-CAD was decreased after knocking down EphA2 or adding 80 μg/mL COE under hypoxic conditions (Figure 5K). Overall, EphA2 is directly regulated by upstream HIF-1a or indirectly regulated by C-MYC under hypoxic conditions, promotes downstream PI3K/FAK phosphorylation, increases VE-CAD expression, and promotes VM formation.

### 3.5 EphA2 promotes HCC VM formation in nude mice

**FIGURE 6:**
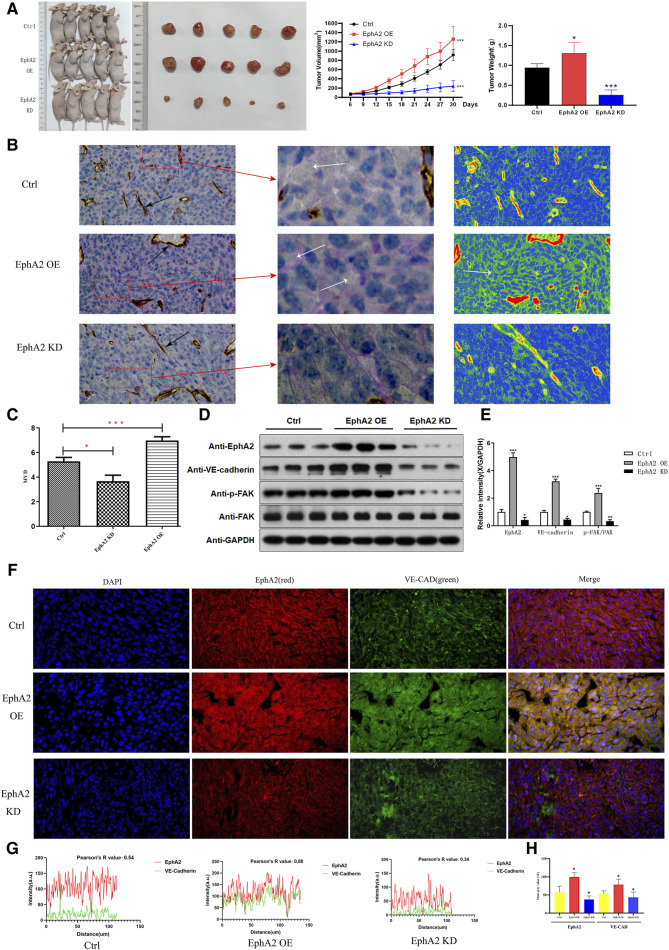
The effect of EphA2 knockdown/overexpression on HCC growth in nude mice. **(A)** Representative images of the subcutaneous tumors formed in nude mice among the control, EphA2-overexpress group and EphA2-knockdown group. Statistical comparison of the difference in tumor volume and weight among the three groups (*p < 0.05, **p < 0.01, ***p < 0.001). **(B)** PAS-CD31 staining of tissue specimen sections of HCC in nude mice. Black arrow: endothelial vessels, White arrow: VM. Thermogram of the internal vasculature of the tumor processed with Casviewer. Red: endothelial vessels. Scale: 50 um. **(C)** The VM formationwas quantitatively counted by micro-vessel density. **(D, E)** Western Blot was used to detect the protein expression levels of EphA2, VE-CAD, p-FAK, FAK after control, EphA2 OE, EphA2 KD treatment. **(F)** The relationship between the expression position and level of EphA2 and VE-CAD in different models of nude mice HCC specimens detected by three-color Immunofluorescence co-localization. (blue: DAPI, green: EphA2, red: VE-CAD, scale: 50 um). **(G)** The mild correlation between EphA2 and VE-CAD in terms of location and level of expression in EphA2 knockdown HCC specimens (*R* = 0.34); significant concordance between location and level of expression in EphA2 overexpressing HCC specimens (R = 0.88). **(H)** The expression of EphA2 and VE-CAD in the overexpression of EphA2 model group washigher than that in the EphA2 knockdown model group in nude mice HCC specimens (*p* < 0.05).

In order to evaluate the role of EphA2 in tumor VM formation *in vivo*, we used MHCC97-H/EphA2 OE and MHCC97-H/EphA2 KD cells to construct xenograft tumor models. The results showed that compared with the control group, the tumor volume and mass were significantly increased in the EphA2 OE group, while the tumor mass and volume were significantly reduced in the EphA2 KD group (Figure 6A). PAS-CD31 staining of tumor tissue showed that VM usually appeared in areas far away from endothelial blood vessels. The VM structure was more abundant after overexpression of EphA2 OE, while it was more difficult to find in EphA2 KD group, and the same phenomenon could be observed in the tumor internal vascular thermogram (Figure 6B). The number of vasculogenic mimicry was counted by quantitative counting of micro-vessel density. The results showed that the average vessel density in the three groups was EphA2 OE > Control > EphA2 KD (Figures 6C, D). The expression level of VM marker (VE-CAD) and the phosphorylation level of PI3K/FAK in EphA2 OE samples were significantly higher than those in the control group, but in the EphA2 KD group, the results were just the opposite (Figure 6E). The three-color immunofluorescence co-localization of the three groups of nude mouse models showed a high degree of consistency between EphA2 and VE-CAD in EphA2-overexpressing tissues (Figure 6F). By analyzing fluorescence intensity, EphA2 correlated with VE-CAD with mild expression in the nude mouse samples of EphA2 KD (*R* = 0.34), whereas there was a significant expression concordance in the EphA2 OE group (*R* = 0.88, Figure 6G). Similarly, the mean fluorescence intensity of EphA2 and VE-CAD in the EphA2 OE group was higher than that in the EphA2 KD group (*p* < 0.05, Figure 6H). In summary, EphA2 is involved in HCC VM formation *in vivo*.

## 4 Discussion

Our previous studies have demonstrated that COE can inhibit the VM formation of HCC *in vitro* and *in vivo* (Jue et al., 2017). It was also found that blocking EphA2 signaling could inhibit HCC VM formation. In this study, by combining network pharmacology with proteomics, we identified EphA2 as a target of COE and an important gene in the progression of HCC. we evaluated the role of EphA2 regulated by COE in the HCC VM formation and the related regulatory mechanisms. EphA2 is closely correlated with VE-CAD, a functional protein for VM formation in HCC. Knockdown and overexpression of EphA2 can affect hypoxia-induced VM formation in HCC cells. Hypoxia can directly regulate the expression of EphA2, or through C-MYC protein, and then through downstream PI3K and FAK to affect the expression of VE-CAD, ultimately affecting VM formation in HCC.

The mechanisms of VM formation are complex and involve interactions between tumor cells and the tumor microenvironment (Treps et al., 2021; Andreucci et al., 2022). Among them, the promotion of VM formation by the hypoxic microenvironment has been confirmed by many studies. In the process of VM formation in HCC, hypoxia-inducible factors can phenotypically transform HCC cells through the LOXL1 pathway and the ROCK pathway, thus promoting VM formation. We observed in CD31/PAS double staining of HCC tissue sections that VM formation was more readily observed in the remote regions than in the adjacent endothelial vasculature areas. The possible explanation for this is that the amount of oxygen that cells can receive decreases with their distance from blood vessels, and cells 200 µm away from blood vessels are essentially in an anaerobic state (Vaupel et al., 2001). However, how hypoxia-inducible factor, regulates the key genes for VM formation in HCC, and which genes play a critical regulatory role in the inhibition of VM formation in HCC by COE, are not known. In this study, we found that knockdown of EphA2 resulted in the inhibition of hypoxia-induced VM formation in HCC cells, while overexpression of EphA2 enhanced VM formation, and partially reversed the inhibitory effect of COE on hypoxia-induced effects. These results suggested that COE exerts its pharmacological effects on the inhibition of VM formation in HCC through EphA2. COE is a mixture obtained by lipid extraction from the stems of *Celastrus orbiculatus*, and the main components are polyterpenoids. Previously, several studies have demonstrated that tretinoin extracted from Tripterygium Wilfordii, another traditional Chinese medicine which belongs to the same genus as *Celastrus orbiculatus*, can exert an inhibitory effect on the VM formation of tumors (Zhang et al., 2021). In addition, some traditional Chinese medicines with the same efficacy description as *Celastrus orbiculatus* may also play a role in inhibiting the tumor VM formation (Jue et al., 2017). Combined with the network pharmacological analyses and the results of proteomic changes after the action of COE on HCC cells, we suggest that the COE inhibit VM formation in HCC by targeting EphA2.

EphA2 was first discovered in human cancers more than 30 years ago (Hirai et al., 1987), Subsequently, EphA2 has been studied in various tumors in numerous studies, and more and more evidence suggests that EphA2 is highly expressed in a variety of tumor tissues, including HCC, and is a key regulator of tumorigenesis and progression, which can modulate a variety of malignant biological behaviors of tumor cells through different signaling pathways. For example, EphA2 can activate the AMPK signaling pathway through the ligand ephrina1-dependent positive pathway and induce breast cancer metastasis (Han et al., 2022). In addition, it has been demonstrated that EphA2 activates ANXA2 by phosphorylating the Tyr426 site of YES1, which in turn promotes the proliferation, migration and invasion of gastric cancer cells (Mao et al., 2021). However, there are few reports on how EphA2 participates in the regulation of VM in HCC under hypoxic condition. As the most important inducer in hypoxic microenvironment, HIF-1a performs multiple hypoxia-induced effects. Analysis of TCGA database revealed that EphA2 and HIF-1a are positively correlated, and JASPAR prediction revealed that HIF-1a had a corresponding sequence to the promoter region of EphA2. The corresponding experiments confirmed that HIF-1a regulates the expression of EphA2. However, the magnitude of this regulation is not obvious, suggesting that there are other regulatory pathways.

It has been reported that HIF-1a/PI3K signaling pathway plays a promoting role in other malignant tumors. For example, Xia et al. (2022) found that bisphenol A produces ROS and activates HIF-1a/VEGF/PI3K/AKT axis through dual-targeting of NADPH oxidase and mitochondrial electron-transport chain, thereby promoting the progression of colon cancer. Han et al. (2022) reported that HIF-1a can induce NID1 expression and activate the PI3K/AKT signaling pathway to promote the metastasis of salivary adenoid cystic carcinoma cells. In addition, Zhang et al. (2018) found that the PI3K/AKT pathway and the expression of VEGF were inhibited after down-regulating HIF-1a, thereby inhibiting the proliferation, migration and invasion of gastric cancer cells. Therefore, we believe that HIF-1a/PI3K may also have some mechanism in the development of hepatocellular carcinoma.

Yoo et al. (2009) reported that the hypoxia-inducible transcription factor HIF-1a was able to inhibit the transcription of DNA repair genes through the atypical mode of action of the “HIF-1a-C-MYC axis.” Tong et al. (2019) demonstrated that cobalt chloride inhibited HepG2 cell growth and induced apoptosis by down-regulating GPC3 expression through the HIF-1a/C-MYC axis. In our proteomics results, we found that the expression of MYC protein showed the same decreasing trend after COE treatment of MHCC97-H cells. Therefore, we hypothesized that C-MYC as a transcription factor has the potential to bind to the EphA2 promoter region to promote its transcription and expression. Corresponding results (Figure 4) finally confirmed that MYC, as a hypoxia-responsive protein, was involved in the regulation of EphA2 expression under hypoxic conditions.

VE-CAD (encoding gene CDH5) protein is involved in the regulation of vascular remodeling and maintenance of vascular integrity. Numerous previous studies have suggested that VE-CAD is a characteristic marker for the transformation of cells from an epithelial phenotype to a mesenchymal phenotype. Williamson et al. (2016) observed co-localization of VE-CAD and VM in small cell lung cancer, the characteristic expression of VE-CAD in SCLC cells forming VM, and the cells no longer possessed the ability to form VM when VE-CAD was knocked down. In this study, we found that there was consistency in the location and intensity of the two staining by PAS staining compared with VE-CAD fluorescence staining. The results of TCGA database analysis suggested there was a linear correlation between EphA2 and VM. Finally, we performed EphA2-VE-CAD fluorescence co-localization assay on HCC tissue sections, and found that there was consistency in the expression amount (same signal) and spatial location (supra/extra-membranous) between EphA2 and VE-CAD, suggesting that EphA2 was expressed simultaneously in the same amount and at the same location as VE-CAD, which also confirmed that there was a direct correlation between EphA2 and VM, thus confirming the direct correlation between EphA2 and VM. Similar phenomenon was also replicated in our mouse model. VE-Cad expression increased after overexpression of EphA2, whereas it decreased significantly after knockdown of EphA2, and the fluorescence co-localization results showed a consistent expression profile of EphA2 and VE-CAD.

This study is a follow-up of our previous research. In supporting our previous report, we further elaborate the role of COE-regulated EphA2 in the VM formation of HCC under hypoxic conditions and some of the molecular regulatory mechanisms. We found HIF-1a directly or indirectly regulates EphA2 through C-MYC, which activates the PI3K/FAK signaling pathway and then promotes the formation of HCC VM. However, hypoxic microenvironment and VM formation are two extremely complex tumor biological phenomena, and the dialogue mechanism between them needs further study. Meanwhile, as a mixture of polyterpenes, the mechanism of action of COE in regulating the growth and metastasis of HCC under hypoxic conditions is not confirmed by the results of this study. In addition, how the inhibition of VM formation affects the effect of anti-angiogenesis on HCC growth and metastasis still needs further exploration. Combination of anti-angiogenesis and immunotherapy is currently the first-line priority choice for locally advanced hepatocellular carcinoma, but the clinical benefits are limited. Whether intervention on EphA2 using natural active ingredients to normalize cancer associated blood vessels can further raise the benefits remains to be further discovered in this study. In the future, we plan to use COE to intervene HCC in animal models and organoids with corresponding EphA2 expression status, in order to obtain more preclinical research data of COE in anti-growth and anti-metastasis.

## 5 Conclusion

**FIGURE 7:**
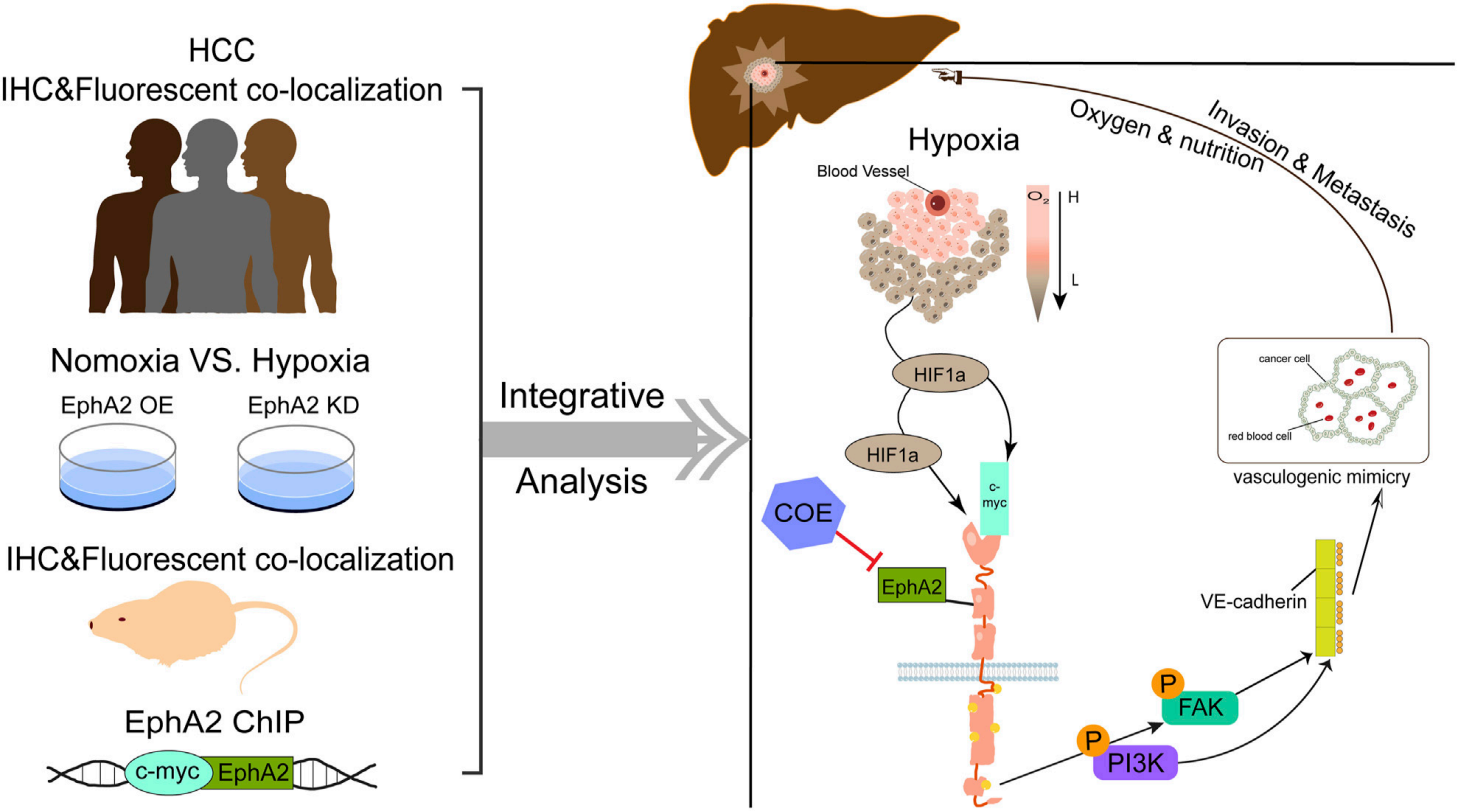
Schematic diagram of study on COE in inhibiting vasculogenic mimicry of HCC.

In summary, we found that COE regulates EphA2 and is involved in the VM formation of HCC by direct regulation by HIF-1a as well as indirect regulation by HIF-1a/MYC, and influences the expression of VE-CAD through PI3K/FAK signaling pathway (Figure 7).

## Data Availability

The original contributions presented in the study are included in the article/Supplementary Material, further inquiries can be directed to the corresponding author.

## Abbreviations

VM, vasculogenic mimicry; HCC, hepatocellular carcinoma; COE, Celastrus orbiculatus extract; EDV, endothelial-dependent vessel; MV, mosaic vessel; EphA2, Erythropoietin-producing hepatocyte receptor A2; CCK-8, Cell Counting Assay Kit-8; GADPH, glyceraldehyde 3-phosphate dehydrogenase; VE-CAD, VE-Cadherin; MMP2, matrix metalloproteinase 2; MMP9, matrix metalloproteinase 9; HIF-1a, hypoxia inducible factor-1a; PI3K, phosphatidylinositol 3-kinase; FAK, Focal adhesion kinase; OE, Overexpress; KD, Knockdown.
